# Phytochemical Targeting of Nerve Growth Factor by Thymoquinone and Cuscutin: A Molecular Dynamics Simulation Study

**DOI:** 10.7759/cureus.63727

**Published:** 2024-07-03

**Authors:** N. Fazulunnisa Begum, Ramya Ramadoss, Pradeep Kumar Yadalam, Pratibha Ramani, Karthikeyan Ramalingam

**Affiliations:** 1 Oral Pathology and Microbiology, Saveetha Dental College and Hospitals, Saveetha Institute of Medical and Technical Sciences, Saveetha University, Chennai, IND; 2 Oral Pathology and Oral Biology, Saveetha Dental College and Hospitals, Saveetha Institute of Medical and Technical Sciences, Saveetha University, Chennai, IND; 3 Periodontics, Saveetha Dental College and Hospitals, Saveetha Institute of Medical and Technical Sciences, Saveetha University, Chennai, IND

**Keywords:** md simulation, cuscuta reflexa, nigella sativa, phytochemicals, molecular dynamic simulations (mds), pain, cuscutin, thymoquinone, oral cancers, nerve growth factor

## Abstract

Background

Nerve growth factor (NGF) is a novel target of pain therapeutics for oral cancer, and it plays a main role in the nociception of chronic pain. Surgery, along with chemotherapy or radiotherapy, is the gold standard for treating patients, but the side effects are significant as well. Newer effective interventions with natural phytochemicals could improve patient compliance and enhance the quality of life among patients with oral cancer. A literature search revealed a positive correlation between NGF and oral cancer pain. *Nigella sativa* (*N. sativa*) and *Cuscuta reflexa* (*C. reflexa*) have proven anticancer effects, but their activity with NGF is unexplored.

Aims and objectives

We aimed to identify the potential phytochemicals in *N. sativa* and *C. reflexa*. We also checked the NGF-blocking activity of the phytochemicals. Molecular docking and molecular dynamic (MD) simulations evaluated the binding energy and stability between the NGF protein and selected phytochemical ligands.

Materials and methods

We obtained protein NGF structure from UniProt (ID: 4EDX, P01138, Beta-nerve growth factor), ligand (thymoquinone) structure using PubChem ID: 10281, and ligand (cuscutin) structure using PubChem ID: 66065. Maestro protein (Schrödinger Inc., Mannheim, Germany) was used for molecular docking. Desmond Simulation Package (Schrödinger Inc., Mannheim, Germany) was used to model MD for 100 nanoseconds (ns). We have assessed the interaction between the protein and ligands by root mean square deviation (RMSD) values.

Results

The interaction of thymoquinone and cuscutin with NGF was assessed. While interacting with thymoquinone, there was mild fluctuation from 0.6 Å to 2.5 Å up to 80 ns and ended up at 4.8 Å up to 100 ns. While interacting with cuscutin, mild fluctuation was seen from 0.8 Å to 4.8 Å till 90 ns and ended at 6.4 Å up to 100 ns. We found a stable interaction between our drug combination and the NGF receptor.

Conclusion

We have identified a stable interaction between thymoquinone, cuscutin, and NGF by our MD simulations. Hence, it could be used as an NGF inhibitor for pain relief and to control tumor progression. Further in vitro and in vivo evaluations of this novel drug combination with phytochemicals will help us understand their biological activities and potential clinical applications in oral cancer therapeutics.

## Introduction

The oral cavity ranks as the 16th most common cancer site in the world, comprising 389,485 new incidence cases and 188,230 deaths according to Globocan Statistics 2022 [1]. The second most common cancer in India is lip and oral cavity cancer. From a histological perspective, oral squamous cell carcinoma (OSCC) accounts for about 90% of oral cancer [1,2]. The overall prevalence of cancer-related pain was more than 50%, with the highest prevalence of about 70% in head and neck cancer patients [3-5]. 

Oral cancer is associated with intense pain during masticatory processes, swallowing, and other functions. Pain in OSCC is the most common symptom, which marks the progression of the potentially malignant lesion to a malignant lesion and is not proportional to the tumor size [3].

Pain control and cachexia are the most challenging symptoms of oral cancer. This pain is well sustained by the secretion of many nociceptive mediators like endothelin-1 (ET-1), proteases, nerve growth factor (NGF), protons, transient receptor potential vanilloid (TRPV), substance P, calcitonin gene-related peptide (CGRP), adenosine triphosphate (ATP), and bradykinin into the cancer microenvironment [6].

NGF is a neurotrophic protein required during the developmental period for neuronal growth, differentiation, and survival. NGF plays a main role in nociception through modulation of inflammatory mediators release, ion channel/receptor activity, and local neuronal sprouting [7]. NGF also has a well-defined role in OSCC pain [8]. NGF also plays a role in cancer cachexia and tumor progression and is highly elevated in OSCC [8,9]. Targeting NGF inhibition in OSCC will reduce nociception, cachexia, and tumor advancement. 

Pain management significantly influences a patient's quality of life, functional ability, and satisfaction with treatment. Therefore, effective pain control can enhance a patient's well-being. The overall survival rate for OSCC patients is approximately 60%, and an improvement in this rate also implies an increase in the pain burden that has to be endured by these patients. They may develop drug tolerance to the most commonly used analgesics, such as opioids [3]. Thus, there is an urgent need for newer alternatives. Currently, the gold standard for treating OSCC patients is surgery combined with chemotherapy or radiotherapy, but these treatments also come with significant side effects. Recently, plant-derived bioactive compounds known as phytochemicals have shown promise in the prophylactic and therapeutic management of various diseases, including cancer [10].

Commonly used herbs, Kalonji with the botanical name *Nigella sativa* (*N. sativa*), and Aftimoon with the botanical name *Cuscuta reflexa* (*C. reflexa*) *Roxb*, are popularly referred to in traditional medicine like Unani and Ayurveda. They have antioxidant and anticancer activity [11,12]. Thymoquinone, the main active compound of *N. sativa*, is widely reported for its anticancer, anti-inflammatory, and immunomodulatory neuroprotective properties [13]. Cuscutin, one of the primary molecules of *C. reflexa*, is also reported to have anti-inflammatory, antioxidant, and anticholinergic effects [14].

Though the *N. sativa* and *C. reflexa* have proven anticancer effects, their activity with NGF is still unexplored. In silico analysis enables a prior understanding of physiological parameters that positively or negatively contribute to drug development. The present study aims to obtain the structures of the selected molecules and to obtain binding energy between the ligand and protein and for molecular dynamic (MD) simulations of the protein receptors and ligands [15]. 

Our goal was to identify potential phytochemicals in *N. sativa* and *C. reflexa*. Additionally, we assessed the NGF-blocking activity of these phytochemicals. We used molecular docking and MD simulations to evaluate the binding energy and stability between the NGF protein and the selected phytochemical ligands.

## Materials and methods

We analyzed the effect of thymoquinone and cuscutin on blocking NGF using MD simulations to evaluate the binding stability and dynamic behavior of the protein NGF and ligand (thymoquinone and cuscutin). Molecular docking analysis data on previously performed phytochemicals of *N. sativa* (thymoquinone, alpha hederin, nigellicine) and *C. reflexa* (cuscutin, kaempferol) were studied. We selected thymoquinone and cuscutin based on a previous study by Begum et al. [15]. 

We obtained the NGF protein structure from UniProt (ID: 4EDX, P01138, Beta-nerve growth factor). The structures of the ligands, thymoquinone, and cuscutin were acquired using PubChem IDs 10281 and 66065, respectively. Molecular docking was conducted using the Maestro software v11.0 (Schrödinger Inc., Mannheim, Germany).

The MD was modeled using the Desmond Simulation Package v4.2 (Schrödinger Inc., Mannheim, Germany) for 100 nanoseconds (ns). We assessed the interactions between the protein and ligands by evaluating the root mean square deviation (RMSD) values.

We used the UniProt (ID: 4EDX, P01138, Beta-nerve growth factor) to retrieve the structure of the protein NGF [16], the PubChem ID 10281 for the ligand thymoquinone C_10_H_12_O_2_, with a molecular weight of 164.2 g/mol, and the PubChem ID 66065 for the ligand cuscutin C_14_H_16_O_9_, with a molecular weight of 328.27 g/mol. Molecular docking was conducted using Maestro proteins (Schrödinger Inc., Mannheim, Germany). The receptor-ligand bond was preprocessed using Maestro’s Protein Preparation Wizard for minimization and optimization. Select ligands were identified in the protein by adding polar hydrogen to amino acid atoms.

Systems were arranged with the System Builder tool, employing the Transferable Intermolecular Potential 3P (TIP3P) solvent model in an orthorhombic box and the Optimized Potentials for Liquid Simulations (OPLS) 2005 force field. Models were neutralized with counterions, and 0.15 M sodium chloride (NaCl) was added to simulate normal physiological conditions.

Desmond is a high-performance MD code for biological systems. MD simulations were done using Desmond for 100 ns. Molecular docking provides a static image of a specific molecule’s binding position with a specific active site on the protein. It is useful for predicting ligand-binding states under static conditions. Docking serves as the initial step in simulating receptor-ligand complexes using MD. MD simulations use Newton’s equation of motion to replicate atomic movements over time, predicting the binding of a ligand to a protein in a stable physiological environment. Simulations were prepared using the isothermal-isobaric (NPT) ensemble at 300 K and 1 atm pressure. The models were equilibrated before the simulation, and trajectories were saved every 100 picoseconds (ps) for analysis. Stability was confirmed by comparing the RMSD values for the ligands and protein over time.

## Results

**Figure 1:**
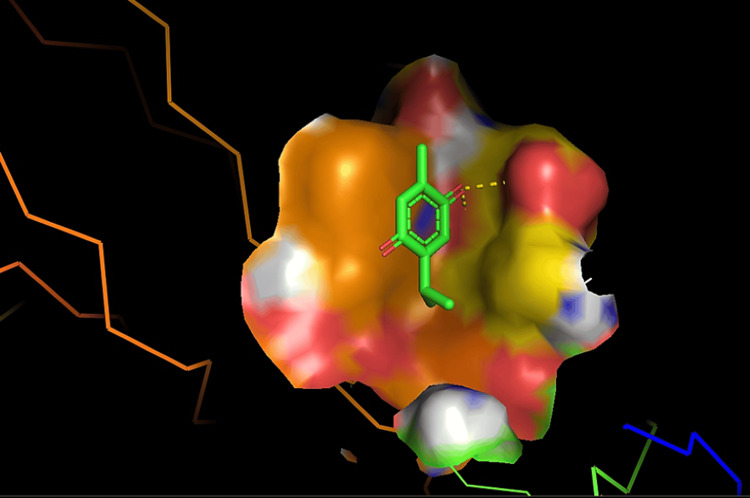
Molecular docking of thymoquinone ligand with nerve growth factor protein

**Figure 2:**
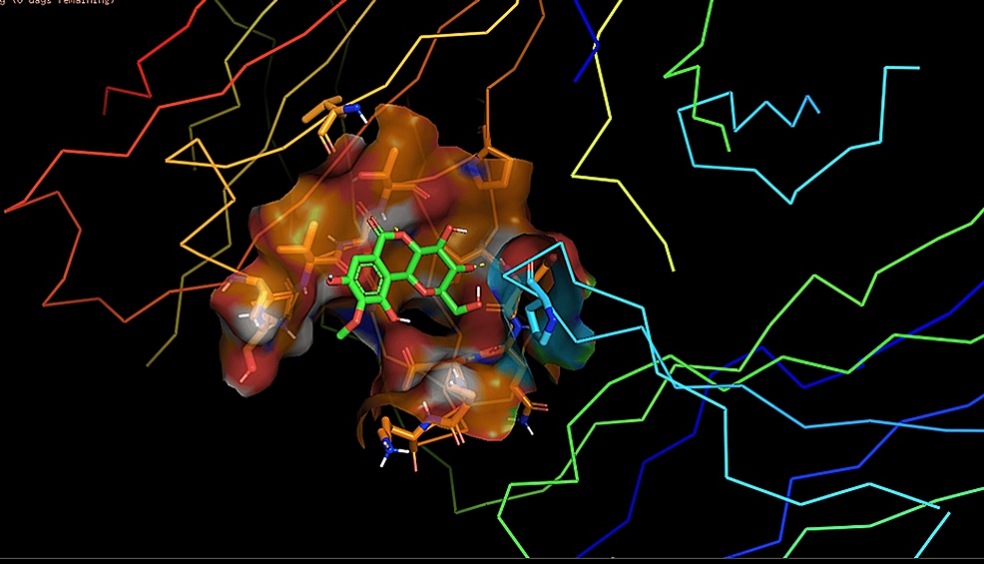
Molecular docking of cuscutin ligand with nerve growth factor protein

Molecular docking of NGF protein structure, thymoquinone, and cuscutin was conducted using the Maestro v11.0 software. During the docking process, the three-dimensional structures of the ligands were computationally fitted into the binding site of the NGF protein to predict their binding affinities and modes of interaction. This involved an in-depth analysis of the conformational flexibility of the ligands and the protein, considering various binding poses and scoring functions to evaluate the most energetically favorable interactions. The interaction of thymoquinone and cuscutin with NGF was assessed using molecular docking and MD simulations (Figures 1-2).

The MD was modeled using the Desmond Simulation Package for 100 ns, with a time resolution of 100 ps for data collection. The dynamics simulations provided insights into the stability and behavior of the protein-ligand complexes over time. We assessed the interactions between the protein and ligands by evaluating the RMSD values, which measured the deviation of the complex structures from their initial configurations, indicating the stability and conformational changes throughout the simulation period. RMSD measured the average distance between the atoms of superimposed proteins or complexes. It provides quantitative insight into the structural stability and conformational changes involving the protein-ligand complex throughout the simulation. By calculating RMSD values for the backbone atoms of the protein and the ligand over time, we could monitor how much the structure deviated from its initial configuration. A low RMSD value indicated that the complex remained stable and close to its original conformation, whereas a high RMSD value suggested significant structural changes or instability. This analysis is crucial for understanding the dynamic behavior and stability of the protein-ligand interactions in a simulated biological environment.

**Figure 3:**
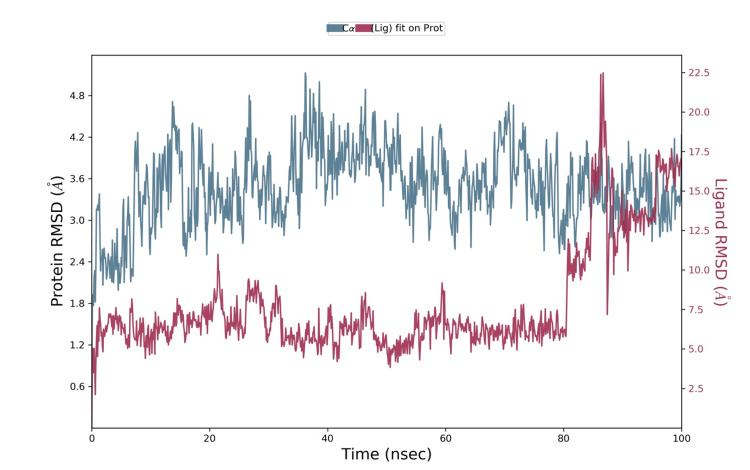
Molecular dynamics simulation obtained from Desmond depicting NGF-thymoquinone RMSD fluctuations NGF: Nerve growth factor; RMSD: Root mean square deviation The quantitative measure of similarity between the two superimposed molecules was represented in the Angstrom unit

While interacting with thymoquinone, there was stability till 80 ns, and mild fluctuation was seen at 80 ns between 6.1 Å and 21.5 Å. Through this stable interaction, we derived that thymoquinone was stable with the receptor (Figure 3).

**Figure 4:**
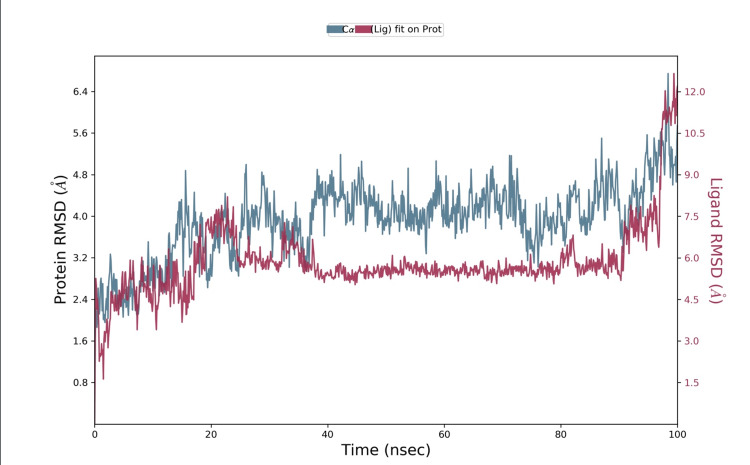
Molecular dynamics simulation obtained from Desmond depicting NGF-cuscutin RMSD fluctuations NGF: Nerve growth factor; RMSD: Root mean square deviation The quantitative measure of similarity between the two superimposed molecules was represented in the Angstrom unit

While interacting with cuscutin, a mild fluctuation was noted for 23 ns between 0.9 Å and 4.2 Å. Then stability was attained at 20 ns, and the interaction was stable till 95 ns. We observed that cuscutin was stable with the receptor (Figure 4).

## Discussion

NGF is a neuroprotein, and targeting it is a complex process. NGF inhibition provides varied benefits in OSCC by reducing pain and inflammation [17,18], reducing tumorigenesis, and aiding in weight loss management [9]. This study is based on the computer-based drug design approach targeting NGF using phytochemicals. NGF is a protein stimulating neurite growth [17]. NGF is a neurotrophin produced by immune cells, endothelial cells, and nerve tissue. A significant effect was observed on inflammation and tumorigenesis, particularly in cancer cells that express NGF receptors on their cell surface [18].

These neurotrophin effects are mediated when binding to the cell-surface receptors like tropomyosin-related kinase receptor/tropomyosin receptor kinase A (TrkA) and p75 neurotrophin receptor (NGFR/p75NTR), of which TrkA is mainly expressed on the cancer cell surface [17]. The phosphoinositide-3-kinase (PI3K)/protein kinase B (Akt) and mitogen-activated protein kinase (MAPK)/extracellular signal-regulated kinase (ERK) are the two main signaling pathways that get altered when NGF binds to the TrkA receptor, thus affecting cell growth, differentiation, and nociceptive effects [18]. 

NGF may also bind to the membrane receptor sortilin, which has a significant role in tumorigenesis [19], and to neuropilin-1 (NRP1), which is necessary for the TrkA signaling of pain [20]. Nociceptive nerves produce neurogenic CGRPs due to the secretion of NGF. NGF secretion is induced by excessive reactive oxygen species (ROS) production by cancer cells, which is associated with excessive pain generation. Also, NGF plays a significant role in cancer stem cell production [17]. Doan et al. [8] stated that the blockade of TrkA and NGF receptors inhibited pain, proliferation, and invasion of OSCC in preclinical and in vitro models. Individual blockade of TrkA controls thermal hyperalgesia, and individual NGFR blockade controls mechanical allodynia. 

It is stated that NGF expression was highly increased in human OSCC tumors and cell cultures. NGF blockade can decrease nociception, restrict tumor proliferation, and reduce weight loss by leptin and pro-inflammatory cytokines production. It has been confirmed that NGF has a common link between pain, proliferation, and cachexia in oral cancer [9]. Anti-NGF could be a probable oral cancer mechanism-based therapy. Increased expression of NGF and tyrosine kinase are considered biological markers for perineural invasion in OSCC [21]. NGF, tumor necrosis factor (TNF)-α, and perineural invasion contribute to the pain and can predict the disease's aggressiveness and prognosis [22]. Targeting both NGF and TNF-α could provide a more enhanced approach to pain management in OSCC [9].

Our present study identified that thymoquinone and cuscutin bind to NGF molecules and have stable interactions, thus blocking or neutralizing the effects of NGF. This could be hypothesized due to the anticancer effects of the two phytochemicals by mainly inhibiting PI3K/Akt pathway activity. Thymoquinone, the main constituent of *N. sativa*, inhibits the proliferation, migration, and invasion and induces apoptosis by inhibiting the PI3K/Akt pathway activity in KB cell lines [23]. Thymoquinone is reported to have modified the PI3k/Akt pathway through reduced phosphorylation, also it leads to upregulation of tumor suppressor protein phosphatase and tensin homolog (PTEN) [24]. It is reported that thymoquinone at different concentrations reduced neuroinflammation by inhibiting TNF-α, IL-1β inflammatory mediators nitric oxide, and prostaglandin E2 (PGE2). Also, it inhibited the production of inflammatory mediators by blocking (PI3K)/Akt/nuclear factor-kappa B (NF-κB) signaling pathway on lipopolysaccharide (LPS)-stimulated BV2 microglial cells [25,26]. Chen et al. showed that thymoquinone inhibited oxidative stress, inflammatory response, and apoptosis through the p13K/Akt pathway [27] in MCF-7 cells, and thymoquinone increased ROS levels, leading to greater phosphorylation of p38-MAPK. When p38-MAPK was silenced, the ability of thymoquinone to induce apoptosis in cancer cells was diminished [28].

A PubMed search using the terms (("thymoquinone"[All Fields]) OR ("cuscutin"[All Fields])) AND (("oral cancer"[All Fields]) OR ("oral squamous cell carcinoma"[All Fields])) did not reveal any published articles. Fath et al. [29] have reported that thymoquinone enhanced cisplatin activity on OSCC cell lines by inducing oxidative stress. Liu et al. [30] have reported the anti-melanogenic effect of cuscutin. Schenck et al. [31] have reported that pro-NGF is detected in higher levels in the oral cavity, and it could be used for analgesia in oral mucositis, recurrent oral aphthous ulcers, and radiation-induced osteonecrosis of jaws, which are frequent side effects of OSCC chemotherapy and radiotherapy. Though our present study exhibits stable interaction between NGF and thymoquinone-cuscutin, further research is needed on the mechanism of action of these phytochemicals derived from *N. sativa* and *C. reflexa* on NGF.

MD simulations offer powerful insights into molecular behavior but have their challenges and limitations. They are computationally intensive, requiring significant resources for large specialized software systems, trained experts, and long timescales. MD simulations are limited to nanosecond to microsecond timescales, often insufficient for capturing slower biological processes. Accuracy depends heavily on force fields, which may not accurately represent all molecular interactions. Simplified models and sampling limitations can lead to biased results, while sensitivity to initial conditions and setup parameters complicates interpretation. MD simulations lack complete predictive power and must be validated with experimental data. Risks of artifacts from boundary conditions and limitations in software further affect accuracy.

## Conclusions

NGF-targeted therapy presents a promising avenue for alleviating chronic pain, mitigating cachexia, and impeding tumor progression in patients with oral cancer. The synergistic potential of combining thymoquinone extracts from *N. sativa* and cuscutin extract from *C. reflexa* warrants further exploration through rigorous in vitro experiments, in vivo studies, and subsequent clinical trials. Investigations must be done to delineate the efficacy and safety profile of these herbal formulations. They offer viable alternatives with potentially fewer adverse effects compared to conventional chemotherapies. Moreover, advancing research in traditional medicine holds promise for enhancing the quality of life among oral cancer patients who endure the burdensome consequences of aggressive interventions. Integrating novel therapeutic strategies from herbal medicine into mainstream oncology will provide complementary treatment options and also address the multidimensional challenges posed by oral cancer. This interdisciplinary approach will connect traditional knowledge with modern scientific validation, potentially creating opportunities for managing symptoms and enhancing patient-reported outcomes in oral cancer care.
